# What Are We Drinking?

**Published:** 1887-02

**Authors:** 

**Affiliations:** Montreal


### WHAT ARE WE DRINKING?

BY AN OCCASIONAL CONTRIBUTOR, MONTREAL.

The old refrain about "the cup that cheers, but not inebriates" passes too often without unfavorable comment. It is greatly to be feared that very few who make use of the apparently innocent and social beverage, suspect the many evils which may lurk under its surface, and the adulteration to which it may have been subjected before it has made its appearance in the "home circle."

On its first introduction into Europe, Britian and America, tea commanded a comparitively high price and was sparingly used, but as the traffic and consumption spread, the demand for it exceeded the possible supply of the genuine article and recourse was had to its imitation in a variety of ways, and spurious counterfeits more or less injurious or fatal to sound health found their way into our home markets. As the demand increases for a greater supply and a *cheaper* article, the counterfeits are multiplied and the adulterations further varied by means of chemicals, coloring, and other spurious imitations.

The compound now called *tea* may be purchased at various prices, from 25 cents upwards, and vast quantities of it are daily consumed which have never known either China or Japan.

It is indisputable that the Chinese do not export their first growth or best quality of tea, but keep it for the use of their Mandarins or high officials and "upper tens." The second quality of Chinese tea goes principally to Russia, where it is greatly in demand and commands a high price, and it is only the inferior and refuse which find their way to England and America. It is a matter of surprise that nations which pride themselves upon their superior intelligence should so long have been the dupes of the cunning Oriental who drives a brisk trade in humbugging the "Melican man."

Missionaries who have lately returned from a residence in Japan as the scene of their lobors, give an account of the tea-packing operations in that country, in which disgusting details are mingled with the ludicrous in about equal proportions. The preparations for export are frequently of inferior qualities, mixed with miscellaneous leaves more or less poisonous. From the foregoing it will be seen that even the greater portion of Chinese and Japanese tea is adulterated, besides being colored with solutions and fluids more or less injurious and poisonous; but many will be "surprised to learn" that there are actually many tea-factories both in England and America. There are no less than eight in the city of London alone, where a compound called "Tea" is made from the exhausted leaves procured from hotels, streets, yards, etc., and then colored with rose-pink, black-lead and other abominations. New York and Montreal also have latterly gone largely into the tea-making business, and new discoveries and inventions made for tinting and adulterating these deleterious compounds, many of which are known only to those who manipulate the business. Large quantities of this vile compound are used in the smaller towns of Canada and the United States.

But even the purest kind of tea, which, by some very unusual chance, may have escaped adulteration of any kind, possesses both *tannic acid* and *theine*, both of which have injurious effects upon the human system, the one being an irritant, and the other an *astringent.* Many of the most eminent medical authorities of the present day have come out boldly in opposition to tea-drinking, giving it as their opinion that it stimulates respiration and causes wakefulness, and although its immediate effect is to increase mental and bodily activity, both are invariably followed by a corresponding depression and exhaustion. Its injurious effects upon the nervous system have also been frequently described, but few realize the extent of the mischief done until their constitutions are shattered beyond remedy by its intemperate use. Its action on the stomach tends to harden and contract this organ, and thus becomes a fruitful source of headache, indigestion, dyspepsia, neuralgia and other wasting and painful nervous diseases. An eminent Canadian physician, in a lecture delivered some years ago in Ottawa, stated that he had examined the remains of a person who had died from the excessive use of tea, and had found the stomach contracted to about *one half* the *natural size.* This lecturer also gave his opinion against the use of tea, recommending in effect that "if you *must* drink tea, you should drink but *little* and that should be of good quality."

It seems also to be very rarely understood or admitted that tea-drinking is a *sensual indulgence* the same as whiskey-drinking, but, of course, with results less marked, and not so immediately dangerous or fatal. Giving up its use, would assuredly be followed in the great majority of cases, by an improved and sounder condition of health, both mental and physical. Although the age moves slowly and gradually in recognizing this fact, there is a decided improvement in this respect within the last few years ; many young persons of both sexes having abandoned tea altogether, substituting in its stead milk, cocoa, chocolate or some of the more nutritious beverages. Of these, milk is the best and most strengthening. Coffee is less injurious, and less liable to poisonous adulteration. The really proper place for tea is as a medicine, and it should be used only as such. France seems to be about the only civilized nation where it is thus most generally rated and used.

Numerous cases might be cited where diseases of many years standing have been cured by the substitution of milk for tea, but there are various other substitutes for tea both palatable and wholesome.

In the large American cities and throughout the west generally, where life is too often one high-pressure of hurry, bustle and turmoil, tea is a leading factor in building up unnaturally high-strung nervous constitutions in which a desire for continuous stimulant seems paramount.

Narratives of pitiful cases are frequently met with in all the leading American papers, where high-born and delicately-nurtured men and women have given way to an apparently insatiable thirst for powerful stimulants. It is so ordered that nature invariably retaliates for any transgression of her laws and suppresses those who persistently disregard them.

Another proof of the powerful effects of tea is found in the well-known fact that tea-tasters, although careful not to swallow the liquid, are obliged, after a short time, to resign their situations, with shattered constitutions.

The following paragraphs from leading authorities on medical matters support the foregoing statements: An article in the "London Medical Times" says:

"Dr. Heath of Newcastle has been the last to raise his voice against tea. But it has long been a fact familiar to us that tea is a most fruitful source of dyspepsia. Among the vast number of poor women who frequent the patient rooms of our London hospitals, we should not be far wrong in saying that two-thirds of them are suffering from dyspepsia. This dyspepsia almost invariably arises from two causes: — the want of proper food, and the abuse af articles like tea, which stay the craving for food, *but which aggravate the consequent conditions of digestion.*"

The "Sanitary Enquirer" of June 1st, 1880, says:

"7000 chests of adulterated tea have been recently burned by order of the British Government. An analysis of many samples taken at random showed 65 per cent. adulteration. In each 100 lbs. there were 65 lbs. of poisonous adulteration. Eleven different poisons were detected, some of them deadly. Probably the worst of Chinese tea comes to America. A large percentage of the stomach pain and indigestion among American women may be traced to tea."

A third paragraph from "Good Health," a magazine published in Battle Creek, Michigan, is as follows: "*All tea is poisonous*: every pound of good tea contains enough *theine*, a poisonous alkaloid, to kill ten men, or at least to make them so sick that recovery would be doubtful; but the natural poisonous properties of tea are greatly increased by the numerous and almost constant adulterations to which it is subjected.

The following is from the "Cincinnati Trade List": "It is well known, at least by grocers and others in the trade, that a great quantity of the tea sold in this country is *absolutely poisonous.* Only a short time ago, in New York City, the sale of over three thousand packages of tea was legally stopped in an auction room, because it was shown that the leaves had been colored by poisonous chemicals, and adulterated to resemble tea of a high grade."

Mr. Herbert Spencer, during his recent visit to America, in the course of a speech delivered in New York City, said: "We hear a great deal about the vile body, and many are encouraged by the phrase to transgress the laws of health. But nature quietly suppresses those who treat disrepectfully one of her highest products, and leaves the world to be peopled by decendants of those who are not so foolish."

Apart from the train of evils already enumerated, tea has a decided tendency to create an unnatural or artificial appetite. It may also be confidently maintained that most of the ills which flesh is heir to are traceable directly to intemperance either in eating or drinking.

As the constant use of stimulants must inevitably tend to weaken and impair health, it is high time that we should give more attention to the quality and quantity of our food and drinks, abandoning that which is hurtful. St. Paul reccommends care of the body, making it imperative on still higher grounds: "Know ye not that ye are the temple of God, and that the Spirit of God dwelleth in you? If any man defile the temple of God, him will God destroy." 1 Cor. III 16, 17.

When given to children or to young persons whose constitutions are in process of growth, tea is hurtful in the extreme. Mothers can scarcely realize that it is to them a slow poison, sapping the foundation of their being by retarding their natural and healthy development, repressing the action of the liver, and building up a high-strung nervous system at the expense of all the vital organs, and forming a constitution so delicate as to be soon broken down.

Those who have grown up from childhood accustomed to its use, in mature years might find it difficult to abandon it at once, but there can be no excuse for those who, knowing its deleterious effects, put the vile slops into the hands of innocent children.

With regard to abandoning the habit of tea-drinking, it may be here remarked that the great majority would experience little or no difficulty in doing so, as it is generally admitted that the giving up of a bad habit is seldom followed by bad consequence, although, of course, to this rule, as to most other general rules, there are exceptions. If the habit has been of long continuance or followed to great excess it would be well to lessen the quantity taken by degrees.

As before remarked, the proper place for tea, would be among medicines, and it is freely admitted that there are frequently cases in which its moderate use may be beneficial, but used as it is now-a-days, freely and constantly, it is undoubtedly highly injurious, and the source of innumerable diseases, attended with much suffering.

